# Effect of Arc Pressure on the Digging Process in Variable Polarity Plasma Arc Welding of A5052P Aluminum Alloy

**DOI:** 10.3390/ma12071071

**Published:** 2019-04-01

**Authors:** Bin Xu, Shinichi Tashiro, Fan Jiang, Shujun Chen, Manabu Tanaka

**Affiliations:** 1 Engineering Research Center of Advanced Manufacturing Technology for Automotive Components, Ministry of Education, Beijing University of Technology, Beijing 100124, China; cougarxbin@163.com (B.X.); sjchen@bjut.edu.cn (S.C.); 2 Joint and Welding Research Institute, Osaka University, Osaka 5670047, Japan; tashiro@jwri.osaka-u.ac.jp (S.T.); tanaka@jwri.osaka-u.ac.jp (M.T.); 3 Beijing Engineering Researching Center of laser Technology, Beijing 100124, China

**Keywords:** VPPA welding, keyhole, digging process, plasma arc pressure, electrode energy balance, X-ray transmission system

## Abstract

The keyhole digging process associated with variable polarity plasma arc (VPPA) welding remains unclear, resulting in poor control of welding stability. The VPPA pressure directly determines the dynamics of the keyhole and weld pool in the digging process. Here, through a high speed camera, high frequency pulsed diode laser light source and X-ray transmission imaging system, we reveal the potential physical phenomenon of a keyhole weld pool. The keyhole depth changes periodically corresponding to the polarity conversion period if the current is same in the electrode negative (EN) phase and electrode positive (EP) phase. There exist three distinct regimes of keyhole and weld pool behavior in the whole digging process, due to the arc pressure attenuation and energy accumulation effect. The pressure in the EP phase is smaller than that of the EN phase, causing the fluctuation of the weld pool free surface. Based on the influence mechanism of energy and momentum transaction, the arc pressure output is balanced by separately adjusting the current in each polarity. Finally, the keyhole fluctuation during the digging process is successfully reduced and welding stability is well controlled.

## 1. Introduction

VPPA (Variable Polarity Plasma Arc) keyhole welding is an ideal method to achieve joints made of middle thick aluminum with high quality and efficiency [1]. There are two polarities in one current cycle: an EN (Electrode Negative) phase and an EP (Electrode Positive) phase. The cycle can achieve both deep penetration and remove the oxide layer on the surface of the base metal [2,3]. The keyhole digging process is very important, because the welding easily fails without proper keyhole generation [4]. However, the evolution of the keyhole and weld pool has not been clearly understood, especially in the digging process of VPPA welding. Therefore, it is necessary to explore the physical phenomenon and the mechanism of the digging process for achieving stable welding.

As a unique characteristic of plasma arc welding, keyhole behavior determines the process stability of plasma arc welding. Keyhole detection research has been carried out in view of DC-PAW (Direct Current Plasma Arc Welding) for steel. Liu et al. [5,6,7] directly captured the keyhole exit image using a CCD (Charge Coupled Device) camera. The keyhole exit deviation distance and keyhole size parameters were put forward to evaluate the thermal state, which provides the feasibility for accurately controlling the keyhole stability. Zhang et al. [8] developed a charge sensor to monitor the plasma cloud dynamics, corresponding to the keyhole status. Metcalfe’s investigation shows that it is possible to indicate whether the keyhole is open or not yet formed by monitoring the efflux plasma. Its fluctuations can reflect the keyhole stability [9]. Regarding to VPPA welding of aluminum, Zheng et al. [10] focused on the front image sensing of the keyhole. An algorithm for extracting the keyhole’s geometrical size was proposed to establish the linkages with weld formation. Wu et al. [11] developed a vision sensor system to acquire the keyhole images. A novel hybrid approach was used to recognize the keyhole status. The prediction is realized through the detection, which is a step forward for accurate control of the keyhole. In addition to directly observing the keyhole with a CCD camera, the unique signal characteristics of VPPA welding were used to measure the keyhole state. Saad et al. [12] achieved identification between three models of VPPA keyhole welding (no-keyhole, keyhole and cutting) using acoustic signal measurement. Wu et al. [13] also investigated the relationship between the keyhole geometry and acoustic signatures with a dual-sensor system. An extreme learning machine model was built for predicting keyhole geometry. However, these methods are mainly used to indirectly obtain the keyhole information. The keyhole boundary inside the weld pool in real time is difficult to measure with the above methods. An X-ray transmission observation system was successfully adopted to investigate the keyhole and weld pool dynamics [14,15,16], which highly contributed to the understanding of keyhole evolution. Anh et al. [17] adopted stereo synchronous imaging of tracer particles with two sets of X-ray transmission systems to clarify the weld pool formation process in DC-PAW.

The above research mainly focused on the keyhole weld pool evolution in a welding quasi-steady state rather than the keyhole digging process. Zheng et al. [18] pointed out that a smooth transition from start-up segment to main body segment was very important for welding stability. By optimizing the waveforms of current and plasma gas flow rate, a relative proper keyhole generation segment was obtained. A synchronous increase of gas flow rate and current contributes to the smooth transition. Chen et al. [19] added a pre-cleaning segment before the parameters increased for easier penetration. The numerical simulation was carried out to reveal the mechanism of the keyhole digging process of VPPA welding. The keyhole weld pool is still prone to be unstable when the welding condition (such as welding position) changes [20,21,22]. Due to the thermal-physical properties of aluminum alloys, welding defects, such as porosity and cutting, are easily generated if the welding process is unstable [23]. Therefore, it becomes very important to understand the plasma arc pressure output and analyze the instability mechanism of the keyhole weld pool. Han et al. [24] measured the plasma arc pressure and analyzed the effect of the arc shape on arc pressure in VPPA. It found that the arc pressure in the EP phase is smaller than in the EN phase when the arc current of different polarities are the same. The existence of a double arc in the EP phase makes the plasma arc pressure reduce further. Jiang et al. [25] measured the VPPA pressure using both pressure transducer and U-tube barometer methods, while the effects of welding parameters were analyzed. The influence of EP on the pressure output is minimal because its time ratio is much less than that of the EN phase. The increase of plasma gas flow rate cools the arc further, resulting in greater constraint of the arc, thus the arc pressure obviously increases. At present, the arc pressure change due to the polarity transaction process is not well understood. Also, the influence of pressure on keyhole weld pool evolution in VPPA welding digging process has not been studied.

Here, we have observed the fluctuation of molten pool surface in the digging process by high speed camera with a high frequency pulsed diode laser light source system. The keyhole boundary in real time was also obtained by an imaging system of an X-ray transmission. In order to analyze the factors influencing the keyhole stability, the plasma arc pressure is measured by the pressure transducer. Combining the energy and momentum balance between electrodes and arc, the physical mechanism of plasma arc pressure was obtained, based on which we optimized the pressure output. Finally, the optimized parameters were verified by the weld formation and weld pool free surface fluctuation situation.

## 2. Materials and Methods

### 2.1. Measurement of Weld Pool Surface Deformation

A5052P aluminum alloy is adopted as the work piece and the chemical composition is in Table 1. The size of aluminum plate is 150 mm × 100 mm × 5 mm.

**Figure 1:**
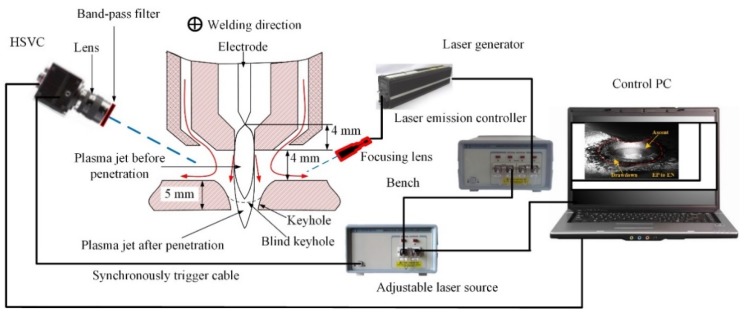
Measurement system of weld pool free surface in digging process of VPPA welding.

The setup for observing weld pool free surface is set as shown in Figure 1. The plasma arc torch includes tungsten electrode, plasma gas nozzle and shielding gas nozzle. The parameters are listed in Table 2. The plasma gas and shielding gas both are argon gas.

In order to clearly observe the weld pool free surface, a telephoto micro lens (AF Micro Nikkor 200 mm, Nikon, Tokyo, Japan) with a 640 nm band-pass filter is used to filter out the arc light, a high-frequency pulsed diode laser light source system (Cavilux HF system, Cavitar, Tampere, Finland) to illuminate the weld pool, a high speed video camera (HSVC) (Memrecam-Q1V, Nac Image Technology, Tokyo, Japan) to record the images. The frame rate of HSVC is 2000 fps. The HSVC sends out an electrical pulse signal to the laser light generator at the start moment of shutter opening, ensuring each picture is clear and reducing the effect of laser heat on the weld pool.

**Figure 2:**
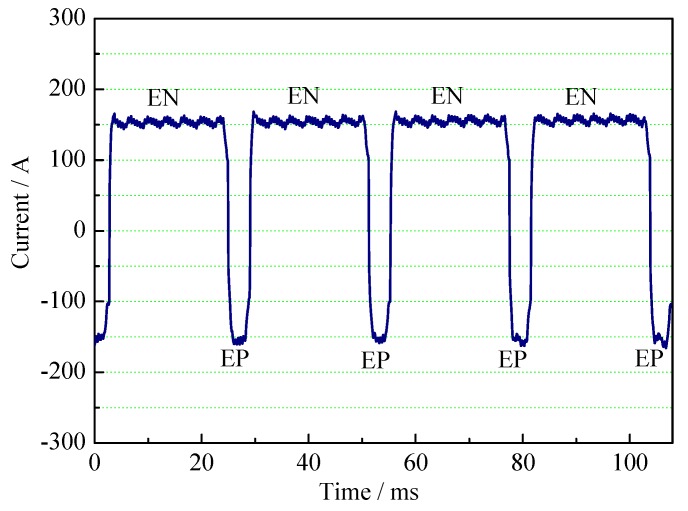
Current waveform of VPPA.

Figure 2 shows the rectangular wave current of VPPA. It is in the EN phase when the current is positive. The duration of the EN phase is longer. The tungsten electrode is connected to the negative pole of power supply while the workpiece is connected to the positive pole. Correspondingly, the current of the EP phase is negative. The duration is shorter. The current flows through the following path: tungsten electrode-workpiece-power supply-tungsten electrode.

### 2.2. Measurement of the Keyhole Boundary during Digging Process of VPPA Welding

**Figure 3:**
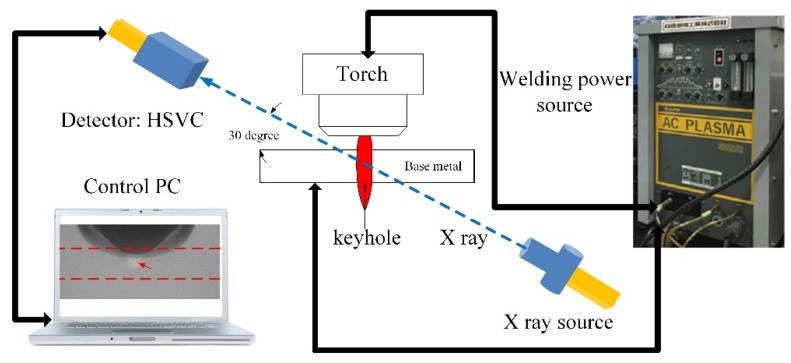
X-ray observation system of the keyhole boundary in digging process of VPPA welding.

**Figure 4:**
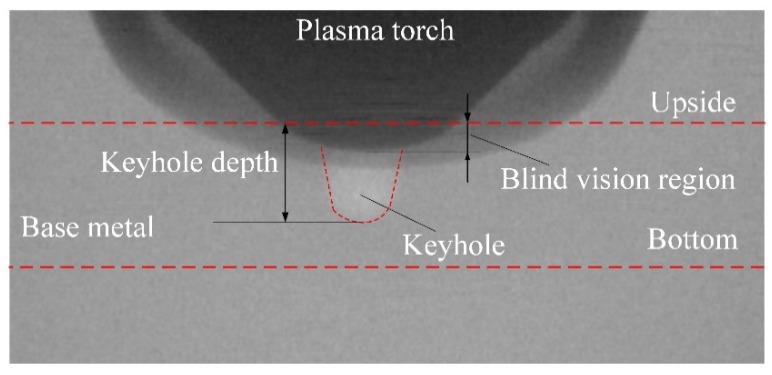
Diagram of the observation results of the small hole boundary.

Figure 3 is the X-ray transmission system used to observe the keyhole boundary in digging process [26]. It consists of one set of X-ray power sources, HSVC and image intensifiers, welding power source and control personal computer (PC). The X-ray power source maximum outputs a tube current of 1.0 mA and a tube voltage of 230.0 kV. In this measurement, X-ray power source is used at 700 µA tube current and180 kV tube voltage. The aluminum plate and parameters are same with that of Section 2.1. As a result, it is difficult for the X-ray to penetrate the entire base metal horizontally. In order to get the relative clear image, the axis line between X-ray source and HSVC is set at 30° with the flat direction to make it easy for X-ray transaction. The X-ray transmission images are recorded by the HSVC at the frame rate of 2000 fps. The camera has an image resolution setting of 800 × 600 pixels to capture a real dimension of 22 mm × 20 mm, as shown in Figure 4. It should be pointed out that due to the angle setting, there is a blind vision region in the observed image. The blind vision region is ignored because of its small size is related to the whole thickness of base metal.

### 2.3. Measurement of Variable Polarity Plasma Arc Pressure

**Figure 5:**
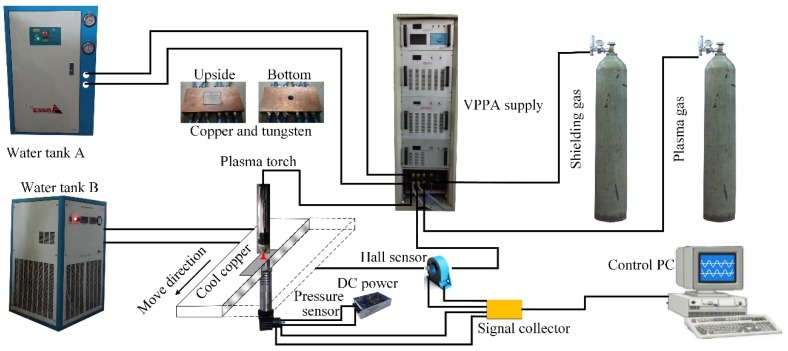
Experimental system of measuring variable polarity plasma arc pressure.

**Figure 6:**
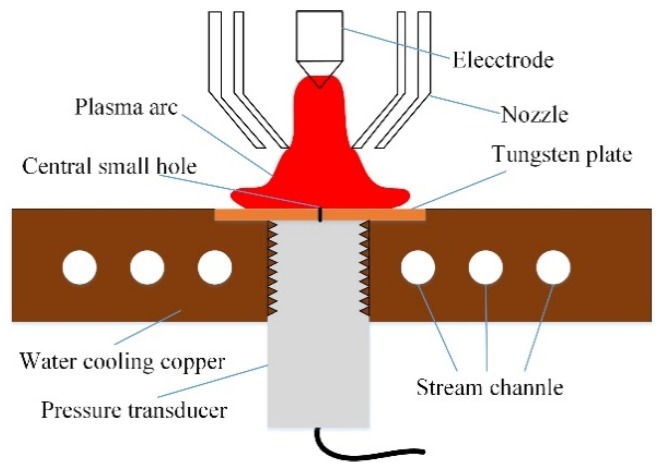
Diagram of measurement structure cross section.

To understand the plasma arc pressure, and thus to analyze its influence on the digging process, the measurement platform is established as shown in Figure 5. Two water tanks are used for cooling plasma torch and the copper, respectively. Arc pressure is measured by the diffusible silicon pressure transducer (Beijing HuaKong xingye technology development Co., Ltd., Beijing, China). The range is from 0 to 5 kPa. A hall sensor is used to get the current synchronously. The measuring frequency is 10,000 Hz. The plane diagram of pressure transducer device is shown in Figure 6. The copper with 6 routes of cooling water is outside. A square tungsten plate is in the center. A small hole with 1 mm diameter is in the center of tungsten plate. Thermally conductive silicone is smeared between the pressure transducer and the cooling copper, which is used to quickly transfer the heat of pressure sensor to the cool copper. The base metal moves with the speed of 1 mm/s for measuring the radial distribution of pressure. The parameters are same with that in Section 2.1. The EP current changes from 150 A to 200 A.

## 3. Experimental Results and Discussion

### 3.1. The Evolution of Keyhole and Weld Pool in Digging Process of VPPA Welding

**Figure 7:**
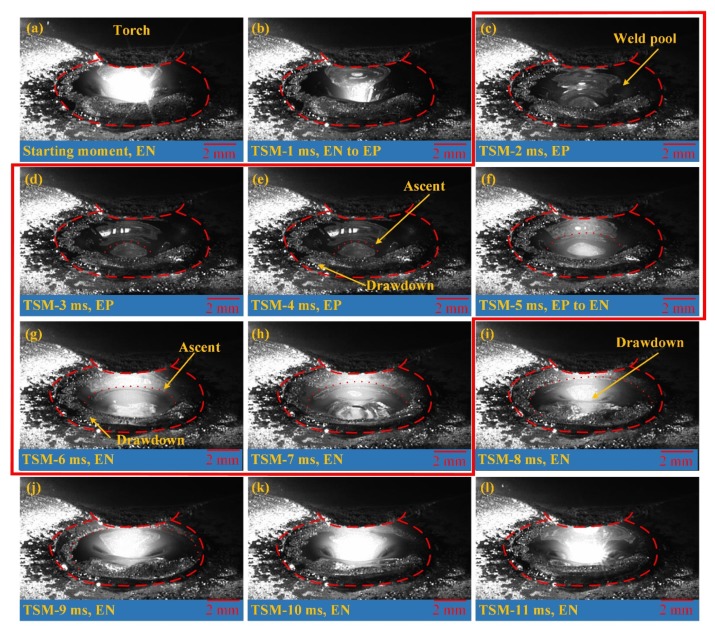
The evolution of weld pool free surface in one current cycle. (**a**) Starting moment (EN); (**b**) TSM-1 ms (EN to EP); (**c**) TSM-2 ms (EP); (**d**) TSM-3 ms (EP); (**e**) TSM-4 ms (EP); (**f**) TSM-5 ms (EP to EN); (**g**) TSM-61 ms (EN); (**h**) TSM-7 ms (EN); (**i**) TSM-8 ms (EN); (**j**) TSM-9 ms (EN); (**k**) TSM-10 ms (EN); (**l**) TSM-11 ms (EN).

In the digging process of VPPA welding, the variation of free surface of weld pool in one current period is analyzed, as shown in Figure 7. The time interval for each picture is 1 ms. The TSM means the time to starting moment. The start moment is the fifth second after arc ignition. One period is separated into four stages: EN phase, EN to EP phase (the polarity switches from EN to EP), EP phase and EP to EN phase (the polarity switches from EP to EN). The disturbance of the weld pool free surface can be obtained from the above four stages. As Figure 7a shows, the weld pool surface in center region concaves while the weld pool edge protrudes up in the EN phase. It does not change when the polarity switches from EN to EP as shown in Figure 7b. After the polarity changes to the EP phase, the weld pool surface in the center rises to the upside. The edge region descends as shown from Figure 7c to Figure 7e. The surface continue to rise up in the EP to EN phase and the start segment of the EN phase is shown from Figure 7f to Figure 7h. Subsequently, the free surface begins to deform rapidly from the center extending outward. The state remains basically stable after TSM-9 ms. Then it moves on to the next cycle and repeats the above phenomenon. The free surface moves up within the red line while it moves down outside the red line.

**Figure 8:**
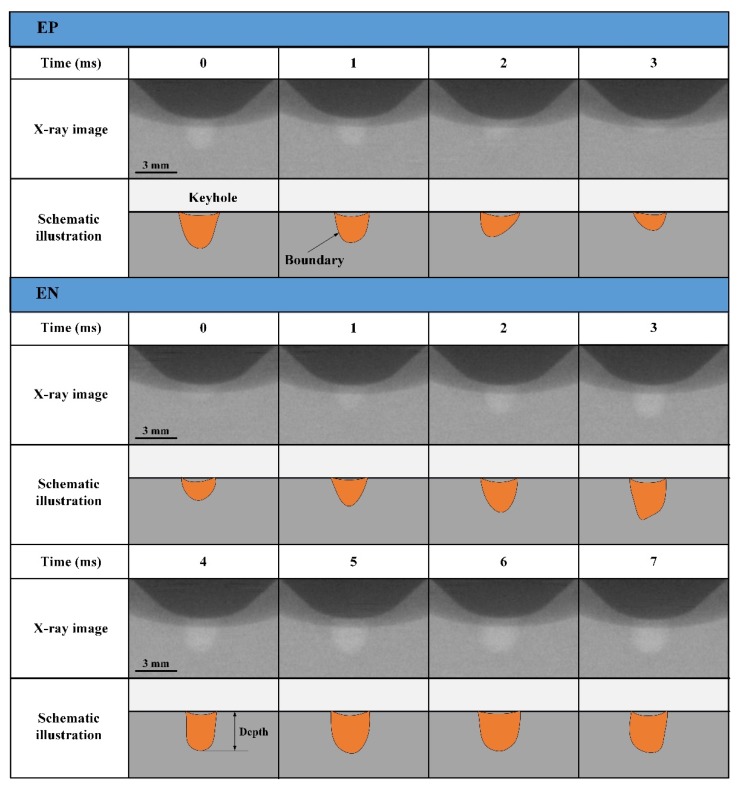
Dynamic characteristics of keyhole during the digging process shown by X-ray imaging technology.

Figure 8 shows the keyhole dynamics measured by the X-ray image system, from which the keyhole boundary inside the weld pool can be obtained. Results in one current cycle (about 8 s from arc ignition) are selected to analyze the keyhole status in different polarity. The schematic illustration is drawn based on the X-ray image. The keyhole size including depth and width obviously reduces from the start of the EP phase to the end. The keyhole size gradually increases from the start of the EN phase as shown from 0 to 3 ms. It remains basically stable when the keyhole increases to a certain size as shown from 4 ms to 7 ms. Through the above observation of the weld pool free surface and keyhole boundary, it is found that there are periodic fluctuations in the state of weld pool during the digging process in VPPA welding for aluminum alloy, which contrasts with the previous study [27,28].

**Figure 9:**
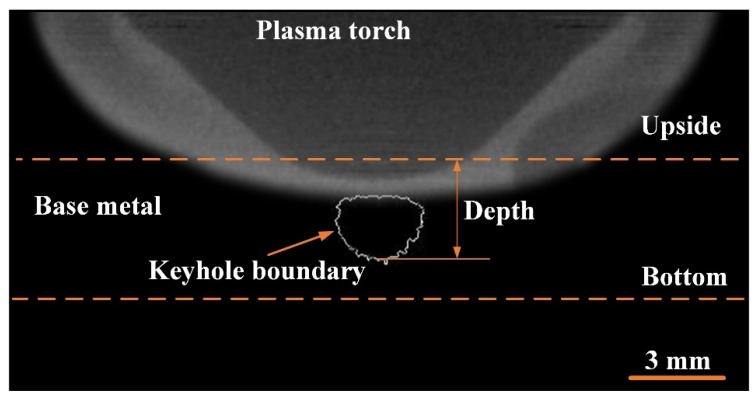
The keyhole boundary indicated by image edge extraction.

The keyhole boundary is obtained by image edge extracting technology based on the X-ray results, as shown in Figure 9. The variation of keyhole depth with time can be quantitatively analyzed by the keyhole boundary results.

**Figure 10:**
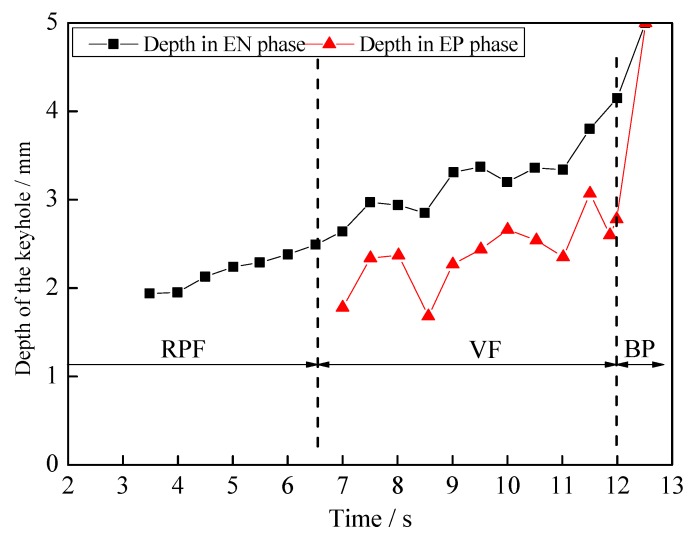
The keyhole depth of a different polarity in the whole digging process.

The keyhole depth with time in the whole digging process is shown in Figure 10. It includes the results of the EP and EN phases, measured depending on the above keyhole boundary. The keyhole digging process can be divided into three distinct regimes of behavior, which are the stages of RPF, VF and BP. The RPF means regular periodic fluctuation, VF means violent fluctuation and BP is blasting penetration, respectively. In the RPF stage, the keyhole depth in the EN phase gradually increases. The keyhole of the EP phase in the RPF stage is in the blind vision region. Combing with the observation results of weld pool free surface, it is considered that the keyhole depth of the EP phase in the RPF stage is close to zero. In the VF stage, the plasma arc in EN phase continually digs the keyhole and the depth increases by about 2.5 mm. Then it gradually increases with a little fluctuation. In this stage, the keyhole of the EP phase appears in the view region and the depth also increases with fluctuation. At the end of the VF stage, the depth of the EN phase is 4.15 mm and that of the EP phase is 2.78 mm. After that it goes into the BP stage and the keyhole is quickly fully established.

**Figure 11:**
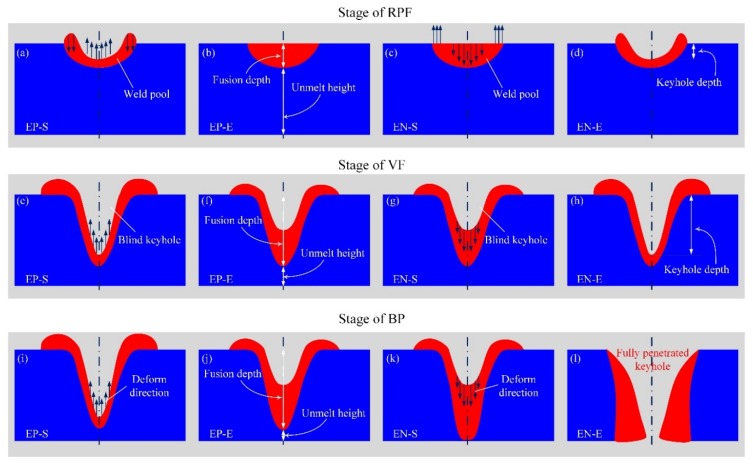
Diagram of keyhole and weld pool in the three stages during digging process. (**a**) start of EP in RPF stage; (**b**) end of EP in RPF stage; (**c**) start of EN in RPF stage; (**d**) end of EN in RPF stage; (**e**) start of EP in VF stage; (**f**) end of EP in VF stage; (**g**) start of EN in VF stage; (**h**) end of EN in VF stage; (**i**) start of EP in BP stage; (**j**) end of EP in BP stage; (**k**) start of EN in BP stage; (**l**) end of EN in BP stage.

The diagram of keyhole and weld pool during digging process is shown in Figure 11. In this figure, EP-S means the start moment of the EP phase. The EP-E means the end of the EP phase. EN-S and EN-E have equivalent meanings for the EN phase. In the stage of RPF, the fusion depth is smaller than the unmelt height, as shown in Figure 11b. The heat of the weld pool is easily transferred out because the unmelt region is large, resulting in a relatively slow melting speed and small size weld pool. Moreover, the plasma arc keeps a strong action on the weld pool if the intensity does not reduce much when the keyhole depth is small. Therefore, in this stage, the response of the keyhole weld pool to the arc status is rapid. Then the plasma arc intensity attenuates seriously with the keyhole depth increase, causing the keyhole weld pool fluctuation, as shown in the VF stage.

In addition, the unmelt height becomes small with the increase of the melting depth, leading to heat loss at the weld pool bottom becoming more difficult. Heat gradually accumulates in the unmelt region. The melting speed gets faster. The keyhole digging speed also increases. Predictably, with the keyhole depth increase, the thermal accumulation also increases. The keyhole digging process and thermal accumulation are mutually reinforcing, resulting in the blasting type penetration in the last stage. Through the above analysis, the keyhole instability during digging process mainly occurs in the first two stages. Adjusting the output of plasma arc pressure to stabilize the keyhole state of EN and EP phases is crucial to realizing stable penetration.

### 3.2. The VPPA Pressure and its Influence Mechanism on Keyhole and Weld Pool Evolution

**Figure 12:**
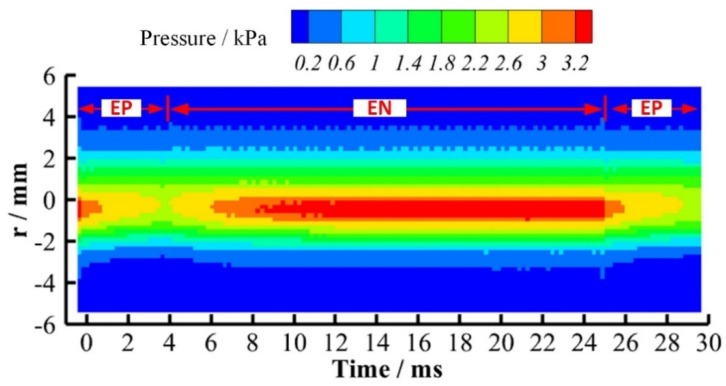
Radial distribution of plasma arc pressure as a function of elapsed time from the beginning of EP phase with the current of 150 A.

**Figure 13:**
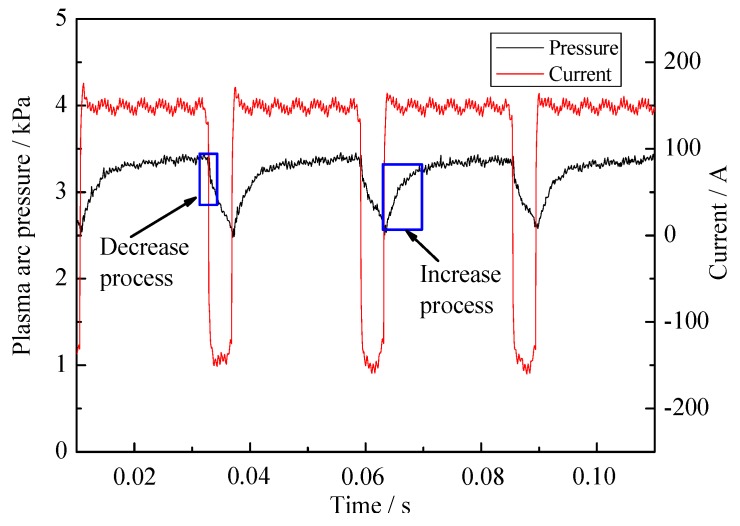
The evolution of plasma arc pressure and current in the center region of arc with 150 A in EN and EP phase.

Figure 12 is the radial distribution of VPPA pressure as the function of elapsed time from the beginning of the EP phase. The currents in EP and EN phase both are 150 A. The pressure is the Gaussian distribution in the radial direction. The radial distribution width of arc pressure has little difference in the EP and EN phases. However, there is an obvious difference between two polarities in the plasma arc center. The pressure of the EP phase is clearly lower than that of the EN phase. Combining with pressure evolution in arc center region shown in Figure 13, we can understand the pressure difference between EN and EP more clearly. In the EN phase, the pressure can keep at a stable value about 3.3 kPa. After the polarity switches from EN to EP, the pressure firstly drops quickly, then gradually decreases. The minimum pressure appears at the moment of polarity switching from EP to EN. Then it increases to the stable value of EN phase, which takes about 6 ms. Therefore, we can conclude that the difference between the two polarities is mainly due to the arc pressure decreasing process and the rising process in the start segment of each phase.

**Figure 14:**
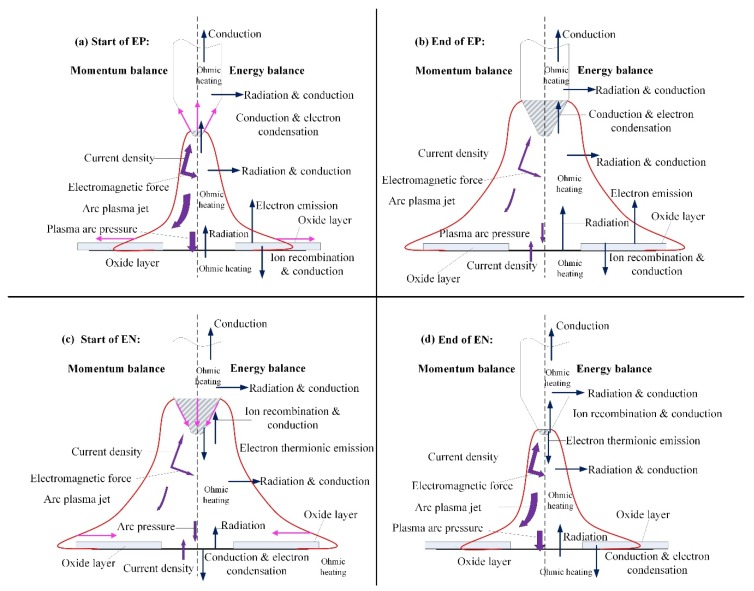
The energy and momentum balance of plasma arc in different polarities. (**a**) start of EP pahse; (**b**) end of EP phase; (**c**) start of EN phase; (**e**) end of EN phase.

In order to understand the evolution mechanism of the VPPA pressure and adjust the pressure output reasonably, the energy and momentum balance between electrodes and plasma arc in different polarities is analyzed in Figure 14. The balance items in the start moment of the EP phase are listed out in Figure 14a, based on which the variation tendencies of the tungsten electrode temperature field and the plasma arc shape are also described, marked by the pink solid line. In the start of the EP phase, the tungsten becomes positive polarity and begins to absorb electrons with the high temperature that comes from the arc column, leading to the temperature of tungsten gradually increasing. Therefore, the melting region becomes larger as shown in Figure 14b. Tanaka et al. [29] synchronously measured the electrode temperature and work function of the tungsten electrode. They found that the work function of the tungsten electrode with the temperature above melting point is obviously lower than that of a solid electrode. Therefore, due to the low work function, the region of current outflow increases. Current density decreases with the increase of the melting region. The plasma arc pressure gradually decreases because of the changing of the current density.

Another reason for the pressure reduction in the EP phase is the different physical process on the interaction of the plasma arc and the base metal in EN and EP phases. Tashiro et al. [30] pointed out that the cathode spot tended to be produced on the oxide layer. The current flowing between the arc and base metal is conducted mainly through the cathode spots. The gradual cleaning of the oxide film from the center to the circumference of the arc also leads to the expansion of the arc shape, causing the current density to decrease further. As shown in Figure 14c,d, the opposite process occurs in the EN phase, which makes the pressure of the EN phase higher.

Moreover, Basins et al. [31] pointed out that the arc pressure can be calculated by the following equation.

(1) $$P_{arc}=\frac{\mu_{0}I^{2}}{4\pi^{2}r^{2}}$$

where $P_{arc}$ is arc pressure, $\mu_{0}$ is the space permeability, *I* is the current, *r* is the radius of plasma arc. Therefore, the difference of pressure between EN phase and EP phase can be got.

(2) $$P_{arc}^{EN}-P_{arc}^{EP}=\frac{\mu_{0}}{4\pi^{2}r_{EN}^{2}}\left(I_{EN}^{2}-\frac{I_{EP}^{2}}{\varphi^{2}}\right)$$

Expressed in terms of current density, Equation (2) gives

(3) $$P_{arc}^{EN}-P_{arc}^{EP}=\frac{\mu_{0}}{4}\left(j_{EN}^{2}r_{EN}^{2}-j_{EP}^{2}r_{EP}^{2}\right)$$

where $P_{arc}^{EN}$ is the plasma arc pressure of the EN phase, $P_{arc}^{EP}$ is the plasma arc pressure of the EP phase, $r_{EN}$ is the radius of the EN plasma arc, $r_{EP}$ is the radius of the EP plasma arc, $I_{EN}$ is the current of the EN phase, $I_{EP}$ is the current of the EP phase, φ is the radius ratio of the EP arc to the EN arc, $j_{EN}$ is the current density of the EN phase, $j_{EP}$ is the current density of the EP phase.

Through the analysis of momentum and energy balance in the interface between electrodes and plasma arc, we can know that the tungsten temperature increase and cleaning of oxide layer on the surface of base metal during EP phase both result in the decrease of current density. Thus, the arc pressure depends not only on the square of the current but also on the square of arc radius based on the Equations (2) and (3). However, Lin et al. [32] pointed out that welding parameters have little effect on the arc pressure distribution radius. Therefore, the best method to balance the pressure of the EP and EN phases is to adjust the current of different phases to change the arc current density separately.

### 3.3. The Optimization of Plasma Arc Pressure and the Molten POOL Stability

**Figure 15:**
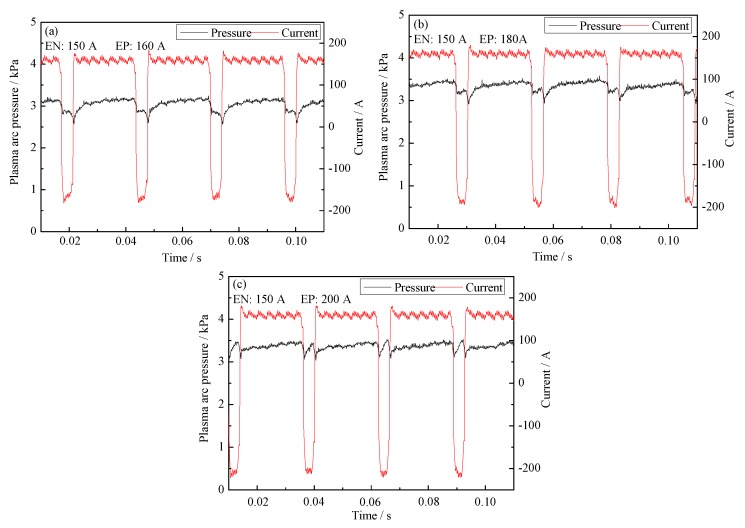
The plasma arc pressure and current waveform with different current of EP phase. (**a**) 150 A in EN phase and 160 A in EP phase; (**b**) 150 A in EN phase and 180 A in EP phase; (**c**) 150 A in EN phase and 200 A in EP phase.

**Figure 16:**
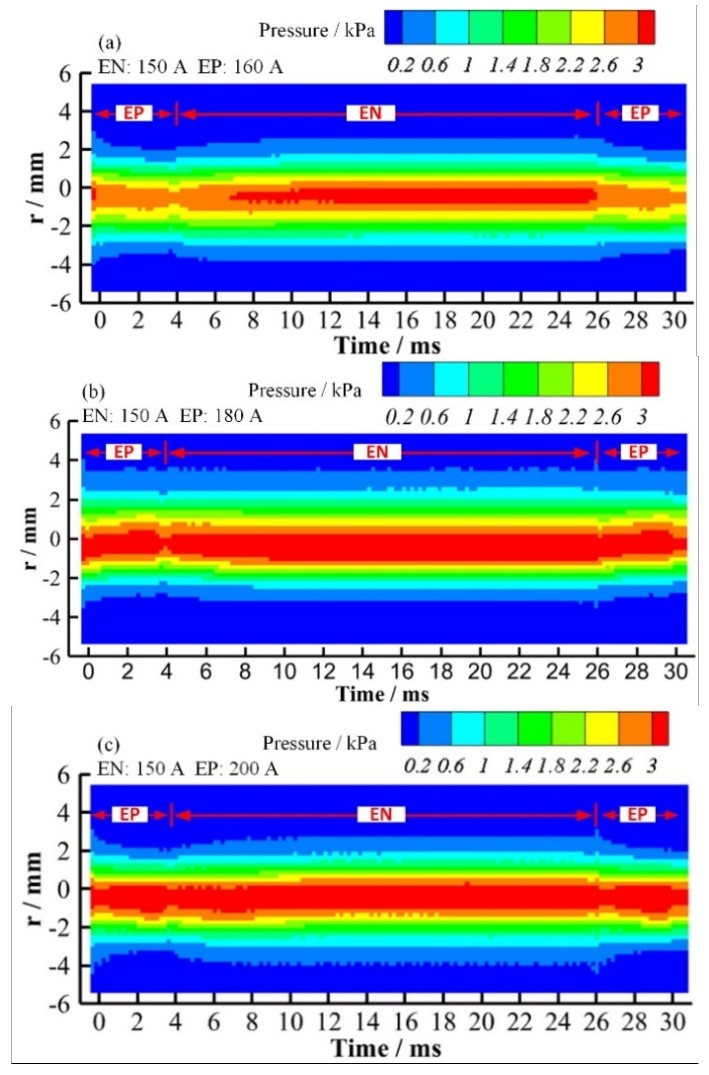
Radial distribution of plasma arc pressure as a function of elapsed time from the beginning of the EP phase with different EP currents. (**a**) 150 A in EN phase and 160 A in EP phase; (**b**) 150 A in EN phase and 180 A in EP phase; (**c**) 150 A in EN phase and 200 A in EP phase.

In this section, the current of the EP phase is adjusted to balance the pressure output. Figure 15 is the evolution of plasma arc pressure in the arc center with a different EP current. The current in the EN phase is fixed to 150 A. The EP current changes from 160 A to 200 A at intervals of 20 A. When the EP current is 160 A as shown in Figure 15a, the pressure of the EP phase is lower than that of the EN phase. The average pressure difference between two phases is around 0.25 kPa. When the EP current increases to 180 A as shown in Figure 16b, the pressure of the EP phase is still lower than that of the EN phase. The pressure between two phases gets closer and the average difference is 0.15 kPa. When the EP current is 200 A, the pressure of the EP phase already becomes a little bigger than that of the EN phase.

The variation of plasma arc pressure with the change in current can be clearly presented by the distribution contour shown in Figure 16. By comparison, it is found that there is no obvious expansion of the arc pressure distribution radius with the changing current. The difference of arc pressure between two polarities is obvious when the EP current is 160 A as shown in Figure 16a. When continuously increasing the current of the EP phase to 180 A and 200 A, the arc pressure of the EP and EN phases tend to be consistent, as shown in Figure 16b,c. Therefore, it can be inferred that the balance of pressure output between the EP and EN phases is achievable by separately adjusting the current in two polarities. Thus, the keyhole weld pool instability during the digging process of VPPA welding can be weakened.

**Figure 17:**
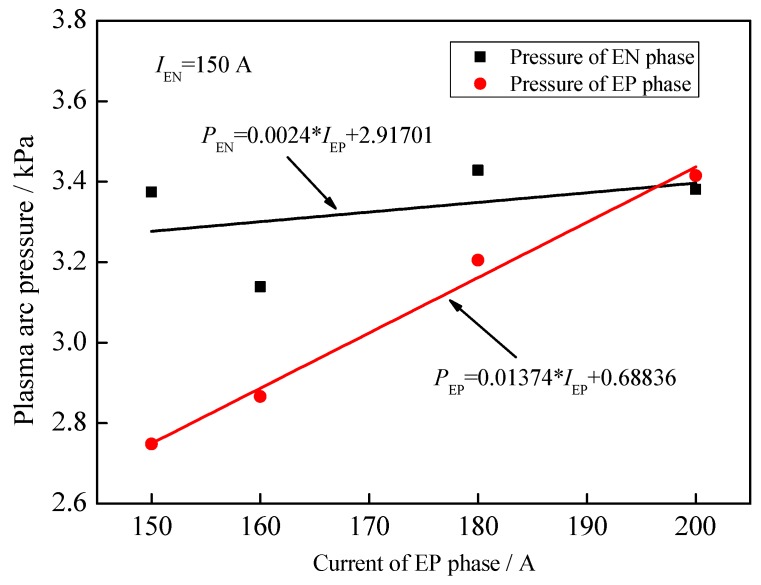
Average plasma arc pressure at different EP currents.

Figure 17 shows the distribution point and fitting line of the average pressure in EP and EN phases with the change of the EP current. The pressure of the EP phase gradually increases and there’s a small fluctuation in the EN pressure. According to the fitting equation, the current of EP phase is 196 A if the pressure of two polarities is equal to each other, when the current of EN phase is 150 A.

**Figure 18:**
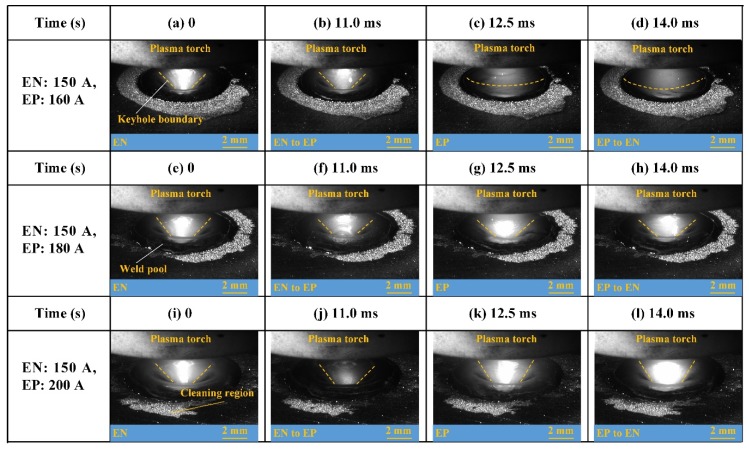
The evolution of weld pool upside surface in one cycle with different EP current: (**a**–**d**) EP = 160 A; (**e**–**h**) EP = 180 A; (**i**–**l**) EP = 200 A.

The weld pool free surface with different EP currents is shown in Figure 18. The influence of balanced pressure output on the weld pool fluctuation is analyzed by comparing the different case. The time 0 in the figures with EN results in around 5 s from arc ignition. Four weld pool statuses in one current cycle are presented: EN, EN to EP, EP, EP to EN, respectively. When the current of EN phase is 150 A and EP current is 160 A as shown from Figure 18a to Figure 18d, the difference of the weld pool free surface between two polarities is obvious. Due to the decrease of plasma arc pressure in the EP phase, the weld pool free surface moves up and close to the upper surface of the base metal. When the current of EP phase is 180 A which is 30 A bigger than that of the EN phase, the difference of free surface deformation in different polarities becomes very small, as shown in Figure 18e–h. When the EP current increases to 200 A, there is also a small weld pool fluctuation between two polarities, which is shown in Figure 17i–l. Therefore, the keyhole and weld pool of the digging process can be stabilized by separately adjusting the current to balance the pressure output.

**Figure 19:**
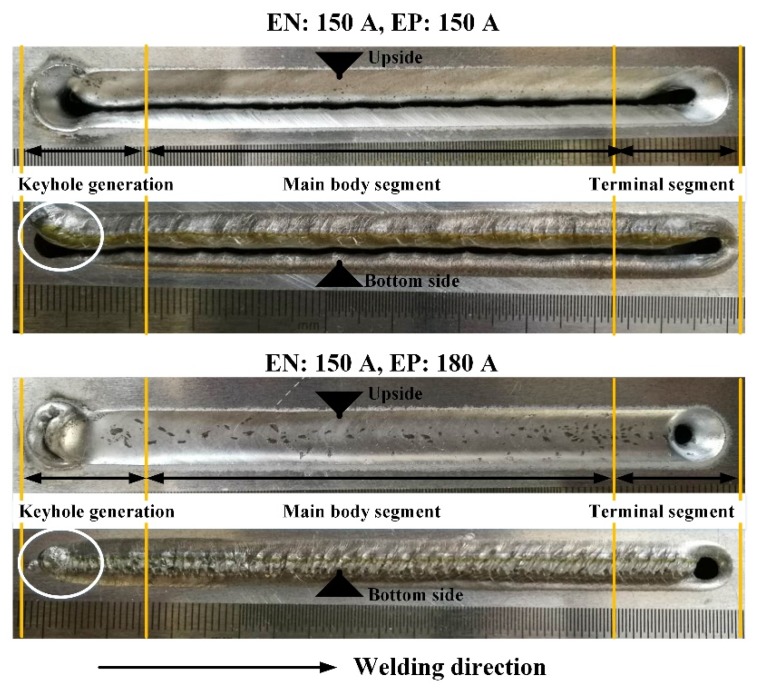
The weld formation with different EP current.

Through a large amount of welding experiments, it is found that the success rate of forming weld bead is low if the keyhole weld pool fluctuates. It is easy to form cutting. As Figure 19 shows, when the current of EN and EP phase is 150 A, all the liquid metal falls to the workpiece bottom in the keyhole generation segment, which makes it difficult to close the keyhole, resulting in cutting. After balancing the pressure output (EN: 150 A, EP: 180 A), the keyhole is more easily closed by avoiding a liquid metal overall drop due to the weak fluctuation. Thus, the weld bead is easier to form.

## 4. Conclusions

This work mainly investigates the stability of keyhole digging process in VPPA welding. Using comprehensive experimental measurement of weld pool free surface, keyhole boundary and plasma arc pressure, the evolution of keyhole weld pool and the influence mechanism of plasma arc pressure are obtained. The specific conclusions are as follows.

(1) The keyhole and weld pool fluctuate with the periodic change of plasma arc state during the digging process of VPPA welding, rather than continuously increasing the keyhole depth over time. By observing the keyhole boundary of digging process in real time, it is found that the keyhole depth increases rapidly in the EN phase, but decreases gradually in the EP phase.
(2) The three stages of keyhole digging process are found, which are regular periodic fluctuation, violent fluctuation and blasting penetration, respectively. The fluctuation of the second stage is caused by the attenuation of plasma arc pressure due to the increase of the keyhole depth. The positive feedback between the thermal accumulation and the continuous increase in the depth of the keyhole leads to the rapid penetration in the third stage.
(3) In order to accurately balance the plasma arc pressure output, the influence mechanism of energy and momentum balance on the pressure is analyzed. The difference between the plasma arc pressure of the EP and EN phases can be effectively reduced if the EP current is 30 A to 50 A larger than that of EN. The pressure balance output can reduce the keyhole weld pool fluctuation in the digging process and improve the welding success rate.

## Figures and Tables

**Table 1:** Chemical composition of A5052P aluminum alloy (wt. %).

Si	Fe	Cu	Mn	Mg	Cr	Zn	Al
<0.25	<0.40	<0.10	<0.10	2.2–2.8	0.15–0.35	<0.10	balance

**Table 2:** Parameters of VPPA welding in digging process.

Process Parameter	Value	Unit
Tungsten diameter	4.8	mm
Nozzle diameter	3.2	mm
Plasma gas flow rate	2.0	L/min
Shielding gas flow rate	10	L/min
Current of EP	150	A
Current of EN	150	A
Duration of EP	4	ms
Duration of EN	21	ms
The standoff	4	mm
Tungsten setback	4	mm
